# Study of Integer Spin *S* = 1 in the Polar Magnet β-Ni(IO_3_)_2_

**DOI:** 10.3390/molecules26237210

**Published:** 2021-11-28

**Authors:** Ebube E. Oyeka, Michał J. Winiarski, Thao T. Tran

**Affiliations:** 1 Department of Chemistry, Clemson University, Clemson, SC 29634, USA; eoyeka@clemson.edu; 2 Advanced Materials Center, Faculty of Applied Physics and Mathematics, Gdansk University of Technology, ul. Narutowicza 11/12, 80-233 Gdansk, Poland; michal.winiarski@pg.edu.pl

**Keywords:** metal iodates, polar magnet, noncentrosymmetry, asymmetric exchange, integer spin

## Abstract

Polar magnetic materials exhibiting appreciable asymmetric exchange interactions can potentially host new topological states of matter such as vortex-like spin textures; however, realizations have been mostly limited to half-integer spins due to rare numbers of integer spin systems with broken spatial inversion lattice symmetries. Here, we studied the structure and magnetic properties of the *S* = 1 integer spin polar magnet β-Ni(IO_3_)_2_ (Ni^2+^, d^8^, ^3^F). We synthesized single crystals and bulk polycrystalline samples of β-Ni(IO_3_)_2_ by combining low-temperature chemistry techniques and thermal analysis and characterized its crystal structure and physical properties. Single crystal X-ray and powder X-ray diffraction measurements demonstrated that β-Ni(IO_3_)_2_ crystallizes in the noncentrosymmetric polar monoclinic structure with space group *P*2_1_. The combination of the macroscopic electric polarization driven by the coalignment of the (IO_3_)^−^ trigonal pyramids along the *b* axis and the *S* = 1 state of the Ni^2+^ cation was chosen to investigate integer spin and lattice dynamics in magnetism. The effective magnetic moment of Ni^2+^ was extracted from magnetization measurements to be 3.2(1) µ_B_, confirming the *S* = 1 integer spin state of Ni^2+^ with some orbital contribution. β-Ni(IO_3_)_2_ undergoes a magnetic ordering at *T* = 3 K at a low magnetic field, *μ*_0_*H* = 0.1 T; the phase transition, nevertheless, is suppressed at a higher field, *μ*_0_*H* = 3 T. An anomaly resembling a phase transition is observed at *T* ≈ 2.7 K in the C_p_/*T* vs. *T* plot, which is the approximate temperature of the magnetic phase transition of the material, indicating that the transition is magnetically driven. This work offers a useful route for exploring integer spin noncentrosymmetric materials, broadening the phase space of polar magnet candidates, which can harbor new topological spin physics.

## 1. Introduction

The lack of spatial inversion symmetry can stabilize asymmetric exchange interactions in systems with unpaired electrons, giving rise to novel physical phenomena such as multiferroics and topological spin textures [1,2,3,4,5,6]. However, engineering noncentrosymmetric (NCS) polar crystal structures for magnetic materials remains difficult, owing to a combination of several factors such as dipole-dipole interaction, steric effect, and thermodynamic effects, which often yield centrosymmetric lattices in transition metal complexes [7,8,9,10]. Polar asymmetric anions with stereo-active lone-pair electrons such as (SeO_3_)^2−^ and (IO_3_)^−^ trigonal pyramids provide avenues for accessing NCS polar structures, facilitated by the second-order Jahn–Teller distortion [6,11,12,13,14,15]. The presence of stereo-active lone-pair electrons and strong spin-orbit coupling from the heavy element (Se, I) in these asymmetric units have been shown to enhance Dzyaloshinskii-Moriya (DM) interaction [3,16].

Increased asymmetric exchange interactions in polar magnets with half-integer spins such as Cu_2_OSeO_3_ (Cu^2+^, d^9^, ^2^*D*), Fe(IO_3_)_3_ (Fe^3+^, d^5^, ^6^*S*), and VOSe_2_O_5_ (V^4+^, d^1^, ^2^*D*) have been demonstrated [2,16,17]; however, studies in integer spin systems featuring the DM interaction have been left unexplored. This is, in part, because there are relatively few integer spin magnets to investigate that have NCS polar lattice symmetries. It has been shown that half-integer and integer spin systems have different magnetic ground states, that is, the former favors a gapless state while the latter has an excitation gap (Haldane gap) between the singlet ground state and the first excited triplet state [18,19,20,21]. An excitation would cost energy in the order of the nearest-neighbor exchange interaction. Here, we study the effects of integer spin in the presence of asymmetric exchange interaction on magnetic properties by looking into the *S* = 1 polar magnet β-Ni(IO_3_)_2_ (Ni^2+^, d^8^, ^3^*F*).

β-Ni(IO_3_)_2_ crystals are twinned by pseudo-merohedry, challenging its structural modelling as well as interpretation of its structure-property relationships [22]. In addition, Ni(IO_3_)_2_ is polymorphic and can condense in hydrate forms depending on atmospheric and synthetic conditions, adding an extra layer of efforts that need to be invested in selectively synthesizing β-Ni(IO_3_)_2_ as well as characterizing the structural and physical properties of this polymorph. The crystal structure of β-Ni(IO_3_)_2_ was previously reported [22]; however, no connections between its chemistry, crystal structure, and physical properties have been established. In this study, we developed a method for synthesizing single crystals and bulk-polycrystalline samples of β-Ni(IO_3_)_2_ that exhibit NCS polar structure with integer spin *S* = 1. We demonstrate that the synthetic chemistry can be used to prepare high-quality bulk polycrystals and single crystalline sample of transition-metal iodates. Furthermore, we probed spin and orbital contribution to the effective magnetic moment of β-Ni(IO_3_)_2_ by performing magnetization measurements. We supplement magnetic properties with specific heat characterization to solidify the thermodynamic signature of the phase transition in this material. This work represents a step forward in accessing atomic engineering of integer spin and breaking inversion symmetry in polar magnets.

## 2. Materials and Methods

Reagents. NiCl_2_ (Alfa Aesar, Tewksbury, MA, USA, 99%), ZnCl_2_ (Alfa Aesar, Tewksbury, MA, UAS, 99.95%), HIO_3_ (Alfa Aesar, Tewksbury, MA, USA, 99.5%), Li_2_CO_3_ (Alfa Aesar, Tewksbury, MA, USA, 99%), and HNO_3_ (BDH, Radnor, OH, USA, 67%) were used as starting materials.

**Figure 1:**
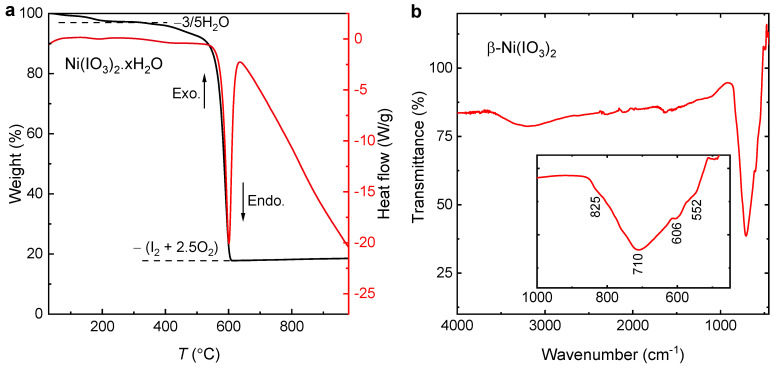
(**a**) Thermogravimetric analysis (TGA) and differential scanning calorimetry (DSC) of β-Ni(IO_3_)_2_/Ni(IO_3_)_2_.2H_2_O showing loss of 3/5H_2_O of crystallization at *T* ≈ 60–200 °C and a decomposition temperature of 523 °C accompanied by the loss of I_2_ and 2.5O_2_. (**b**) FTIR of β-Ni(IO_3_)_2_ showing four absorption bands corresponding to four fundamental I–O vibrations expected for IO_3_ group in *C*_3v_ symmetry *Γ*_vib_ = 2*A*_1_ + 2*E*.

Synthesis of β-Ni(IO_3_)_2_. Polycrystalline β-Ni(IO_3_)_2_ was obtained by heating a mixture of β-Ni(IO_3_)_2_ and Ni(IO_3_)_2_.2H_2_O at 300 °C for 8 h (Figure 1). For β-Ni(IO_3_)_2_/Ni(IO_3_)_2_.2H_2_O mixture, NiCl_2_ (2.5 mmol) was dissolved in HNO_3_ (1 M, 25 mL), an aqueous solution of HIO_3_ (5 mmol, 10 mL) was added, and the solution was stirred at 80 °C for 1 h. A green solution was obtained and allowed to evaporate completely in a fume hood. A yellow-brown solid, subsequently determined to be β-Ni(IO_3_)_2_, was obtained after 4 days (Yield, 68%).

β-Ni(IO_3_)_2_ single crystals were grown by hydrothermal reaction. Li_2_CO_3_ (1 mmol), NiCl_2_ (1 mmol), and H_2_O (4 mL) were placed in a 23 mL Teflon-lined autoclave. The autoclave was heated at 200 °C for 96 h and cooled slowly to 25 °C at a rate of 5 °C/h. β-Ni(IO_3_)_2_ yellowish crystals of were isolated by filtration and washed with deionized water.

Synthesis of Zn(IO_3_)_2_. ZnCl_2_ (2.5 mmol) was dissolved in HNO_3_ (1 M, 25 mL). An aqueous solution of HIO_3_ (5 mmol, 10 mL) was added, and the mixture was stirred at 80 °C for 1 h to give a colorless solution. Zn(IO_3_)_2_ white solid precipitated from the solution after 3 days and was collected by filtration and dried in air (yield of Zn(IO_3_)_2_, 65%; based on Zn).

Single Crystal X-ray Diffraction. Single crystal diffraction experiments were performed on β-Ni(IO_3_)_2_ using a Bruker D8 Venture diffractometer with Mo Kα radiation (λ = 0.71073 Å) and a Photon 100 detector at *T* = 300 K. Data processing (SAINT) and scaling (SADABS) were performed using the Apex3 software system. The structure was solved by intrinsic phasing (SHELXT) and refined by full matrix least-squares techniques on F2 (SHELXL) using the SHELXTL software suite. All atoms were refined anisotropically.

Powder X-ray diffraction. Powder X-ray diffraction (PXRD) measurements on polycrystalline β-Ni(IO_3_)_2_ and β-Ni(IO_3_)_2_/Ni(IO_3_)_2_.2H_2_O mixture were performed using the Rigaku Ultima IV diffractometer equipped with Cu Kα radiation (λ = 1.5406 Å). Data were collected in the 2*θ* range of 5–90° at 0.2°/min scan rate. Rietveld refinement of XRD pattern was performed using TOPAS Academic V6. Vesta software was used for crystal structure visualization [23].

Synchrotron X-ray Diffraction. Synchrotron XRD patterns of β-Ni(IO_3_)_2_/Ni(IO_3_)_2_.2H_2_O mixture were collected using the 11-BM beamline at Advanced Photon Source, Argonne National Laboratory. Data were collected at *T* = 295 K and *λ* = 0.45789 Å.

Thermal analysis. A TA SDT Q600 Instrument was used in the thermogravimetry (TG) and differential scanning calorimetry (DSC) measurements. Approximately 10 mg of the solid was isolated from the synthesis, and the β-Ni(IO_3_)_2_/Ni(IO_3_)_2_.2H_2_O mixture was placed in an alumina crucible and heated at a rate of 20 °C/min from room temperature to 1000 °C under flowing nitrogen (flow rate: 100 mL/min).

Infrared spectroscopy. The attenuated total reflection Fourier transform infrared (ATR-FTIR) spectrum for β-Ni(IO_3_)_2_ was collected using a Shimadzu IR Affinity-1S in 400 to 4000 cm^−1^ frequency range.

UV-Vis spectroscopy. Optical reflectance was measured on β-Ni(IO_3_)_2_ bulk powder using Agilent Cary UV–Vis (NIR) 7000 Universal Measurement Spectrophotometer from 2500 nm to 350 nm. A mixture of approximately 20 mg of β-Ni(IO_3_)_2_ and 100 mg of BaSO_4_ was pelletized and used for the measurement.

Magnetization and specific heat. Magnetization measurements on β-Ni(IO_3_)_2_ powder were performed with the vibrating sample magnetometer (VSM) option of a Quantum Design Physical Properties Measurement System (PPMS). Data were collected under the applied magnetic fields of *μ*_0_*H* = 0.1 T and 3 T from *T* = 2 K–340 K. Magnetic susceptibility was approximated as magnetization divided by the applied magnetic field: *χ* ≈ *M*/*H*. Heat capacity was measured using the PPMS, employing the semiadiabatic pulse technique from *T* = 2 K–300 K.

## 3. Results and Discussion

Polycrystalline anhydrous β-Ni(IO_3_)_2_ has a brownish color, which is distinct from the green color of hydrated Ni(IO_3_)_2_.2H_2_O, while β-Ni(IO_3_)_2_ single crystals have a yellow color. The β-Ni(IO_3_)_2_:Ni(IO_3_)_2_.2H_2_O ratio of the precursor mixture was determined to be 71%:29% by Rietveld refinements of powder XRD data (Figure S1). Pure β-Ni(IO_3_)_2_ powder was obtained by heating β-Ni(IO_3_)_2_/Ni(IO_3_)_2_.2H_2_O mixture at *T* = 300 °C. The annealing temperature was determined from the thermogravimetry (TG) and differential scanning calorimetry (DSC) experiment (Figure 1a). In TG/DSC analysis, the mass reduction of 2.8% at *T* ≈ 60–200 °C corresponds to the loss of ~3/5H_2_O. β-Ni(IO_3_)_2_ is thermodynamically stable at *T* = 220–350 °C, suggesting that *T* = 300 °C is an appropriate reaction temperature for the synthesis. The decomposition of β-Ni(IO_3_)_2_ starts at *T* = 523 °C, accompanied by a strong endothermic transition in the DSC curve. The mass reduction is attributed to the loss of I_2_ and 2.5O_2_ (experimental mass loss = 79.4%; calculated mass loss = 79.6%). The polymorphic behavior of Ni(IO_3_)_2_ was previously studied by Raman and FTIR spectroscopy [24]. Based on the spectroscopic evidence, β-Ni(IO_3_)_2_ can be converted to α-Ni(IO_3_)_2_ or Ni(IO_3_)_2_.2H_2_O, depending on temperature, time, and atmospheric condition. We observed the conversion of ~23% of β-Ni(IO_3_)_2_ to Ni(IO_3_)_2_.2H_2_O over the course of approximately 6 months. This transition was confirmed by the synchrotron X-ray diffraction experiment (Figure S2).

### 3.1. Crystal Structure

**Figure 2:**
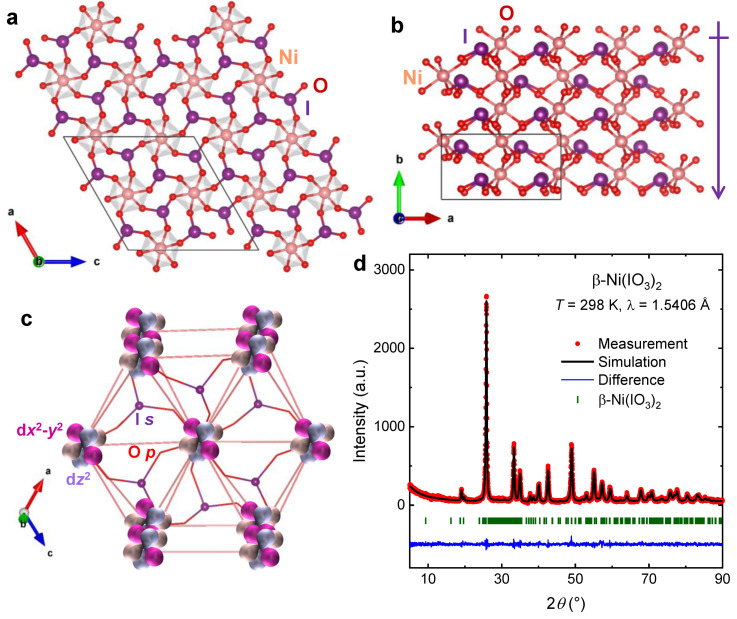
(**a**,**b**) Crystal structure of β-Ni(IO_3_)_2_ consisting of corner-sharing NiO_6_ octahedra and IO_3_ trigonal pyramid; the polar symmetry of β-Ni(IO_3_)_2_ results from the alignment of (IO_3_)^−^ local dipole along the *b* axis. (**c**) Atomic connectivity and orbital overlap in β-Ni(IO_3_)_2_. (**d**) Rietveld refinements of XRD data of polycrystalline β-Ni(IO_3_)_2_.

To confirm the reported structure of β-Ni(IO_3_)_2_ [22] and evaluate its phase purity, we performed single-crystal and powder X-ray diffraction experiments. Details of the crystal structure of β-Ni(IO_3_)_2_ derived from single crystal X-ray diffraction are summarized in Table 1. The room-temperature powder XRD pattern of β-Ni(IO_3_)_2_ is in excellent agreement with the structure obtained from single crystal X-ray diffraction (Figure 2).

β-Ni(IO_3_)_2_ crystallizes in the NCS polar monoclinic structure with space group *P*2_1_. The crystal structure of β-Ni(IO_3_)_2_ can be described as a 3D network of corner-sharing NiO_6_ octahedra and (IO_3_)^−^ trigonal pyramids. The unit cell of this material comprises two nonequivalent Ni^2+^ positions, and each Ni^2+^ cation is bonded to six oxygen in a slightly distorted octahedra, with Ni–O bond lengths ranging from 2.03(3) Å to 2.16(4) Å. Each I^5+^ in (IO_3_)^−^ unit is bonded to three oxygen atoms in a trigonal pyramidal geometry, induced by the stereo-active lone-pair electrons of the I^5+^ cation (s^2^) [12,13]. The I–O bond lengths range from 1.76(3) Å–1.85(4) Å. The subtle distortion of NiO_6_ octahedra is likely induced by the packing effect of the (IO_3_)^−^ asymmetric building units [7,12,16].

The polar symmetry of β-Ni(IO_3_)_2_ results largely from the alignment and addition of the (IO_3_)^−^ local dipole along the *b* axis (Figure 2a) [12,13]. FTIR spectra of β-Ni(IO_3_)_2_ show four absorption bands corresponding to four fundamental I–O vibrations expected for the (IO_3_)^−^ group in *C*_3v_ symmetry *Γ*_vib_ = 2*A*_1_ + 2*E* (Figure 1b). The combined effect of the broken inversion symmetry, macroscopic polarization, stereo-active lone-pair electrons, and triangular sub-lattice of the Ni^2+^ cations can facilitate competing magnetic exchange interactions in β-Ni(IO_3_)_2_ [25,26,27].

Ni(IO_3_)_2_.2H_2_O crystallizes in the centrosymmetric orthorhombic lattice with *P*bca space group, and the crystal structure comprises a 2D network of corner-sharing trans-NiO_4_(H_2_O)_2_ octahedra and (IO_3_)^−^ trigonal pyramids [28]. Unlike in β-Ni(IO_3_)_2_, the (IO_3_)^−^ trigonal pyramids in Ni(IO_3_)_2_.2H_2_O are arranged in opposite directions along the *b* axis, giving rise to the centrosymmetric structure with zero polarization.

### 3.2. UV–Vis Spectra

**Figure 3:**
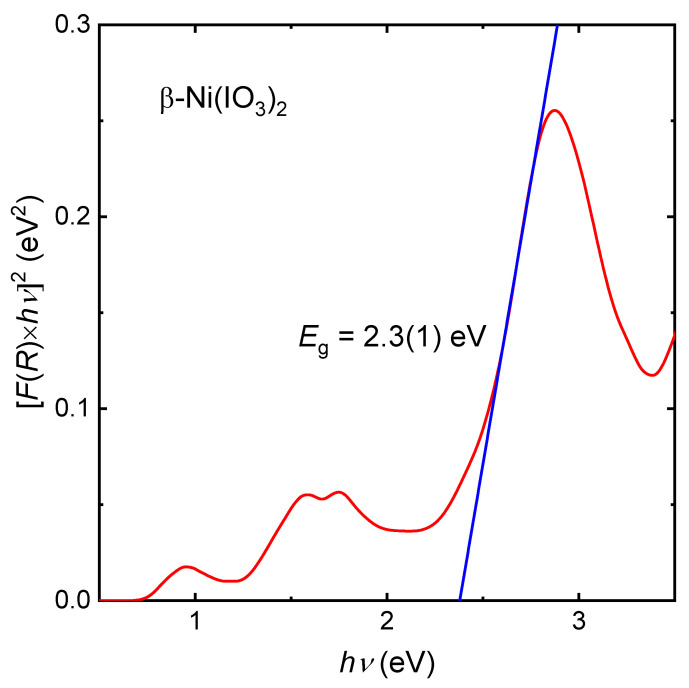
Tauc plot for β-Ni(IO_3_)_2_ showing characteristics of the electronic transitions and estimated optical bandgap of 2.3(1) eV.

**Figure 4:**
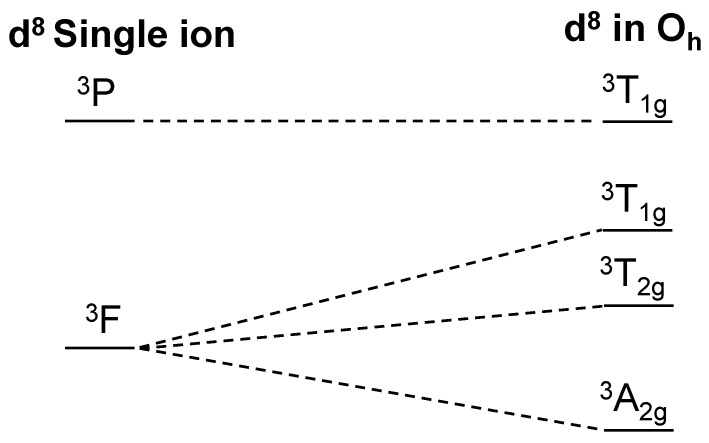
Energy states for a d^8^ ion in O_h_ symmetry based on the ligand field theory.

To study the electronic transitions and estimate the optical bandgap of this material, we performed UV–Vis reflectance measurement on β-Ni(IO_3_)_2_ powder. Figure 3 shows features of three characteristic bands associated with d → d electronic transitions of a d^8^ metal cation in octahedral geometry. Energy states for a d^8^ ion in O_h_ symmetry is presented in Figure 4. The bands at 0.85, 1.6, and 1.8 eV are ascribed to ^3^T_2g_ ← ^3^A_2g_, ^3^T_1g_(F) ← ^3^A_2g_ and ^3^T_1g_(P) ← ^3^A_2g_ electronic transitions, respectively. The optical bandgap was estimated from the linear region of the Tauc plot of [*F*(*R*) *× hν*] versus *hν* to be 2.3(1) eV (Figure 3) [29]. This is consistent with the brown-yellow color (2.1–2.3 eV) of the compound.

### 3.3. Magnetization

**Figure 5:**
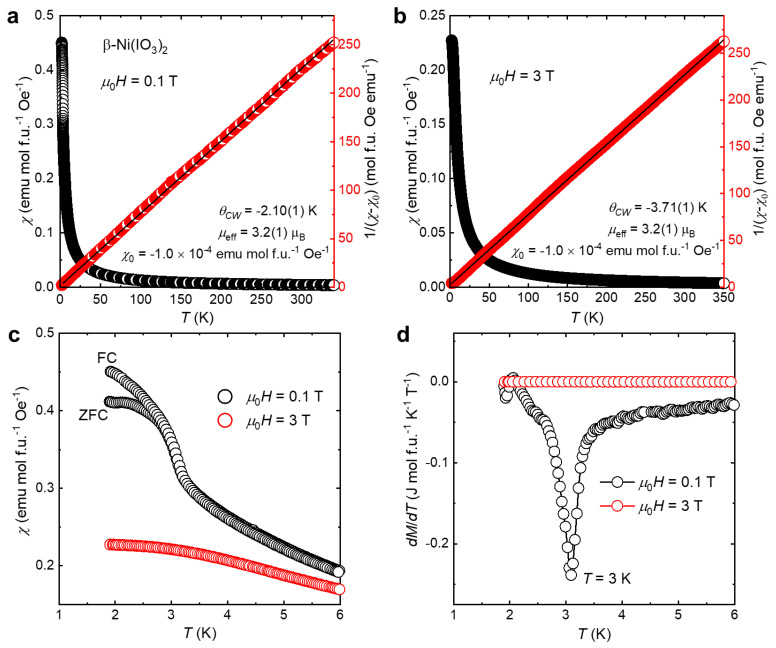
(**a**,**b**) (Black) magnetic susceptibility of β-Ni(IO_3_)_2_ measured from *T* = 2 K–300 K at (**a**) *μ*_0_*H* = 0.1 T and (**b**) *μ*_0_*H* = 3 T. (red) Curie–Weiss fitting of 1/(*χ* − *χ*_0_) against temperature for the paramagnetic phase. (**c**) Zero-field-cooled (ZFC) and field-cooled (FC) magnetic susceptibilities of β-Ni(IO_3_)_2_ as a function of temperature at different magnetic field. (**d**) First derivative of magnetization with respect to the temperature, *dM*/*dT* vs. *T* for β-Ni(IO_3_)_2_.

To evaluate the magnetic properties of β-Ni(IO_3_)_2_, we performed temperature-dependent magnetization measurement from *T* = 2 K–300 K at different magnetic fields *μ*_0_*H* = 0.1 T and 3 T (Figure 5). High density data of dc magnetization were collected using the VSM option of PPMS to enable accurate estimation of magnetic transition temperature and phase change. β-Ni(IO_3_)_2_ exhibits field-dependent magnetic behavior. At low magnetic field *μ*_0_*H* = 0.1 T, β-Ni(IO_3_)_2_ undergoes a phase transition at *T* = 3 K, while at high magnetic field *μ*_0_*H* = 3 T, no magnetic phase transition was observed. Magnetic susceptibility curve above the magnetic phase transition was fit to the Curie–Weiss equation (Equation (1)):

(1) $$\chi (T)=\frac{C}{T-\theta_{CW}}+\chi_{0},$$

where *C* is the Curie constant, *θ_CW_* is the Curie–Weiss temperature, and *χ*_0_ is the temperature-independent contribution to the susceptibility, which includes the small diamagnetic signals of the electron core and the sample holder [30].

The effective magnetic moment *µ*_eff_ per Ni^2+^ cation was estimated using the relation (Equation (2))

(2) $${µ}_{\mathrm{eff}}=\sqrt{\left(\frac{3k_{B}}{N_{A}}\right)}C,$$

where *N_A_* is the Avogadro number, and *k*_*B*_ is the Boltzmann constant. The obtained effective magnetic moment of β-Ni(IO_3_)_2_ at both magnetic fields is 3.2(1) µ_B_, which is slightly higher than the ideal spin-only magnetic moment *g*(*S*(*S*+1))^1/2^ = 2.83 μ_B_ expected for a free spin *S* = 1. This result is consistent with the *S* = 1 integer spin state with some orbital magnetic moment. Unlike the pseudo 1D Haldane SrNi_2_V_2_O_8_ [21], the *χ*(*T*) plots of this material do not show a typical signature of low dimensional magnetic systems (a broad hump over a wide temperature range); these curves nevertheless exhibit a rather flat feature at 100 K < *T* < 350 K. This suggests that the magnetic exchange interactions of β-Ni(IO_3_)_2_ are not 1D but in higher dimensions of spin communication network. This observation is supported by the proposed 3D orbital overlap of β-Ni(IO_3_)_2_ (Figure 2c), which pertains to its electronic dimensionality and thus its magnetic interactions. Neutron diffraction experiments would provide additional insights into these magnetic exchange couplings.

An inverse susceptibility vs. temperature plot highlights the Curie–Weiss character of the magnetic susceptibility of β-Ni(IO_3_)_2_ (Figure 5a,b). The Curie–Weiss temperature *θ*_CW_ of β-Ni(IO_3_)_2_ was extracted from the intercept of the linear fit to be −2.1 K and −3.7 K at *μ*_0_*H* = 0.1 T and 3 T, respectively. The negative values of the Curie–Weiss temperature signify antiferromagnetic (AFM) exchange interaction [31], and the small values can indicate either weak AFM coupling or competing exchange interactions. The zero-field-cooled (ZFC) and field-cooled (FC) magnetic susceptibilities as a function of temperature measured at applied magnetic fields *μ*_0_*H* = 0.1 T and 3 T are presented in Figure 5c. ZFC and FC susceptibilities show a bifurcation below the transition temperature and a downturn at ~2.5 K, demonstrating the possibility of competing AFM-FM interactions at low magnetic field [31,32].

The field-dependent magnetic properties of β-Ni(IO_3_)_2_ can be further illustrated in the plot of first-derivative of magnetization as a function of temperature *dM*/*dT* vs. *T* at different fields (Figure 5d). An apparent peak is observed at *T* = 3 K and *μ*_0_*H* = 0.1 T, indicating magnetic phase transition from a paramagnetic state to a magnetically ordered state. At *μ*_0_*H* = 3 T, no phase magnetic phase transition is observed.

**Figure 6:**
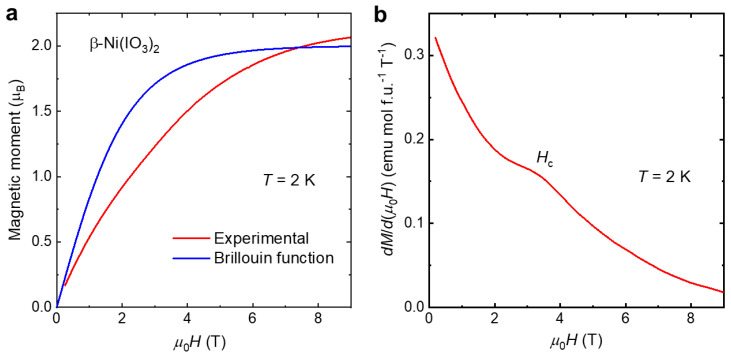
(**a**) (red) *M*(*H*) data of β-Ni(IO_3_)_2_ in μ_B_ at *T* = 2 K, (blue) Brillouin function for *J* = 1 and *T* = 2 K. (**b**) *dM*/*dH* curve for β-Ni(IO_3_)_2_, showing critical field *H*_c_ at ~3 T.

To gain more insight into the nature of the magnetic phase transition, we measured the evolution of magnetization as a function of magnetic field at *T* = 2 K (Figure 6). The nonlinear character of the *M*(*H*) curve cannot be simply described. In Figure 6a, Brillouin function (blue) describes magnetization of non-interacting paramagnetic spins, and the *M*(*H*) data of β-Ni(IO_3_)_2_ are shown in μ_B_ for comparison. The *M*(*H*) curve should saturate at around 2(g*J), but instead, it crosses the 2.0 limit and reaches a higher saturation moment at a higher field, indicating orbital contribution to the magnetic moment. This is consistent with the results extracted from the Curie–Weiss analysis illustrated above. In addition, the deviation from the Brillouin function curvature likely suggests some AFM interactions present in the system. A critical field of ~3 T is observed in the *dM*/*dH* curve as a function of magnetic field (Figure 6b). *dM*/*dH* declines and approaches zero at high magnetic fields, suggesting either saturation of magnetization or formation of a singlet state. Nevertheless, additional studies are needed to provide better understanding of the magnetic ground state of the material.

### 3.4. Heat Capacity

**Figure 7:**
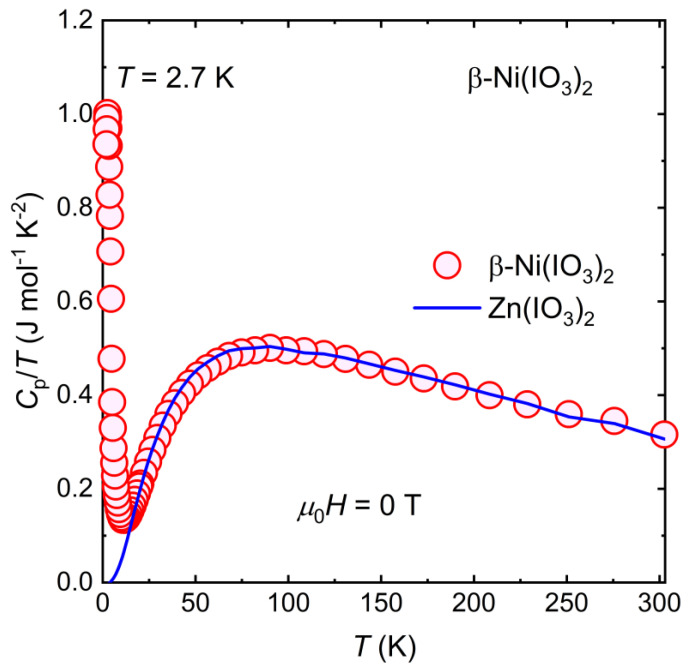
Molar heat capacity over temperature (*C*_p_/*T*) vs. temperature (*T*) for β-Ni(IO_3_)_2_.

To investigate the thermodynamic properties of the ground state of this *S* = 1 integer spin polar magnet, we performed a zero-field specific heat capacity measurement over a temperature range of 2 K ≤ *T* ≤ 300 K (Figure 7).

An anomaly is observed at *T* ≈ 2.7 K in the *C*_p_/*T* vs. *T* plot, which is approximately the temperature of the magnetic phase transition in β-Ni(IO_3_)_2_. The entropy recovered ∆S from this transition can be estimated from Equation (3):

(3) $$\Delta S=\int_{0}^{T}\frac{C_{v}}{T}dT,$$

where *C_v_* is the heat capacity at constant volume, which is approximated as *C*_p_—heat capacity at constant pressure—for solid at low temperatures, and *T* is the temperature. Phonon contribution to specific heat entropy can be accurately estimated using appropriate nonmagnetic isostructural material. The heat capacity of nonmagnetic isostructural Zn(IO_3_)_2_ was used to subtract the phonon contribution. The entropy recovered from the specific heat data at 2 K ≤ *T* ≤ 300 K was estimated to be 3.3(1) J mol f.u.^−1^ K^−1^, which is ~36% of the expected value of *R*ln(3) for *S* = 1 (Figure S3). The entropy change accompanying the magnetic phase transition in β-Ni(IO_3_)_2_ was underestimated because the transition was not complete down to *T* = 2 K. Further specific heat measurements at *T* < 2 K in the presence of applied magnetic fields will enable a more complete elucidation of the thermodynamic properties of the magnetic ground state of β-Ni(IO_3_)_2_.

## 4. Conclusions

Studies in polar magnetic materials possessing asymmetric exchange interactions, which are relevant to possible emergence of topological spin physics, have been in a rather confined space of half-integer spins attributed to the scarcity of integer spin systems with broken spatial inversion symmetry. β-Ni(IO_3_)_2_ was selected for the focus of this work due to its combined integer spin *S* = 1 and NCS polar lattice components. The synthetic approach for both single crystal and polycrystalline form of β-Ni(IO_3_)_2_ developed in this study provides facile chemistry for preparing new polar magnetic compounds. The magnetic behavior of β-Ni(IO_3_)_2_ is field-dependent, i.e., the material undergoes a phase transition at *T* = 3 K at low magnetic field, but the transition is suppressed at higher fields. The phase transition is magnetically driven, as evident by an anomaly observed at approximately the same temperature in the specific heat data. Taken together, the results provide us with an avenue for expanding the phase space of polar magnets to integer spins. Additional ac magnetization, specific heat measurements, and neutron diffraction could help further demonstrate the magnetic ground state at zero filed and magnetic states at finite fields, as well as determine whether integer spins in a polar crystal lattice have any impact on tuning asymmetric exchange interactions and thus potential vortex-like spin textures.

## Figures and Tables

**Table 1:** Crystal structure information and refinement parameters for β-Ni(IO_3_)_2_ obtained by single crystal X-ray diffraction.

Refinement Parameter	Crystallographic Data
Chemical formula	Ni(IO_3_)_2_
Color	Yellow
Shape	Block
Size (mm × mm × mm)	0.02 × 0.02 × 0.02
Formula weight (g/mol)	408.51
Temperature (K)	300
X-ray radiation	Mo *K*_α_
Wavelength (*λ,* Å)	0.71073
Crystal system	Monoclinic
Space group	*P*2_1_
Z	4
*a* (Å)	10.8067(4)
*b* (Å)	5.1190(2)
*c* (Å)	10.8151(4)
*α* = *γ* (°)	90
*β* (°)	119.7950(10)
*V* (Å^3^)	519.20(3)
*ρ*_calc_ (g/cm^3^)	5.226
No. of reflections collected	7093
*μ* (mm^−1^)	15.582
2*θ* (°)	55
GOF	1.141
Flack	0.07(5)
*R*(*F*) *^a^*	0.051
*R_w_(F_o_*) *^b^*	0.1046

*^a^*$R\left(F\right)=\sum ||F_{o}|-|F_{o}^{2}||/\sum |F_{o}|$. *^b^*$R_{w}\left(F_{o}^{2}\right)={\left[\sum w{\left(F_{o}^{2}-F_{o}^{2}\right)}^{2}/\sum w{\left(F_{o}^{2}\right)}^{2}\right]}^{1/2}$.

## Data Availability

Data supporting reported results can be sourced directly from the authors.

## Supplementary Materials

The following are available online, Figure S1: Rietveld fit of powder XRD pattern of the bulk polycrystalline β-Ni(IO_3_)_2_ precursor-β-Ni(IO_3_)_2_/Ni(IO_3_)_2_.2H2O mixture. Figure S2: Rietveld fit of synchrotron XRD pattern of the bulk polycrystalline β-Ni(IO_3_)_2_ after 6 months in air. Figure S3: (a) Molar heat capacity over temperature (*C_p_*/*T*) vs. temperature for β-Ni(IO_3_)_2_, (b) Magnetic entropy change from 2 K to 300 K, ∆S_mag_ (2 K ≤ T ≤ 300 K) = 3.3(1) J mol^−1^ K^−1^~36% of expected value for S = 1 spins (Rln3). Table S1: Crystal structure information and refinement parameters for β-Ni(IO_3_)_2_ obtained by Rietveld refinement of powder X-ray diffraction patterns. Table S2: Atomic positions for β-Ni(IO_3_)_2_ obtained from the single crystal XRD. Table S3: Bond lengths and bond angles of β-Ni(IO_3_)_2_. Table S4: Calculated bond valence sum (Vi) of β-Ni(IO_3_)_2_.

## Sample Availability

Samples of the compounds β-Ni(IO_3_)_2_ and Zn(IO_3_)_2_ are available from the authors.
