# Supplementary material for: Study of Integer Spin S = 1 in the Polar Magnet β-Ni(IO3)2
Source: Molecules. 2021 Nov 28;26(23):7210. doi: 10.3390/molecules26237210 (PMC8658994; doi:10.3390/molecules26237210)
Supplement: Supplementary file 1 [file molecules-26-07210-s001.zip › molecules-1426689-supplementary.pdf]

# Supporting Information

## Study of Integer Spin S = 1 in the Polar Magnet β-$Ni(IO_3)_2$

Ebube E. Oyeka,[1] Michał J. Winiarski,[2] and Thao T. Tran*,[1]

[1]Department of Chemistry, Clemson University, Clemson, SC 29634, USA

[2]Faculty of Applied Physics and Mathematics and Advanced Materials Center, Gdansk University of Technology, ul. Narutowicza 11/12, 80-233 Gdansk, Poland

Corresponding Author: thao@clemson.edu

**Figure S1.** Rietveld fit (black line) of powder XRD pattern of the bulk polycrystalline β-$Ni(IO_3)_2$ precursor - β-$Ni(IO_3)_2$/$Ni(IO_3)_2.2H_2O$ mixture (red points) and the blue line is the difference between the measured data and the simulation.

**Figure S2.** Rietveld fit (black line) of synchrotron XRD pattern of the bulk polycrystalline β-$Ni(IO_3)_2$ after 6 months in air (red points) and the blue line is the difference between the measured data and the simulation.

**Figure S3.** (a) Molar heat capacity over temperature ($C_p/T$) vs. temperature for β-$Ni(IO_3)_2$. (b) Magnetic entropy change from 2 K to 300 K, $\Delta S_{mag}$ (2 K $\leq T \leq$ 300 K) = 3.3(1) J mol$^{-1}$ K$^{-1}$ ~ 36 % of expected value for $S = 1$ spins ($R$ln3).

**Table S1.** Crystal structure information and refinement parameters for β-$Ni(IO_3)_2$ obtained by Rietveld refinement of powder X-ray diffraction patterns.

**Table S2.** Atomic positions for β-$Ni(IO_3)_2$ obtained from the single crystal XRD.

**Table S3.** Bond lengths and bond angles of β-$Ni(IO_3)_2$.

**Table S4:** Calculated bond valence sum ($V_i$) of β-$Ni(IO_3)_2$.

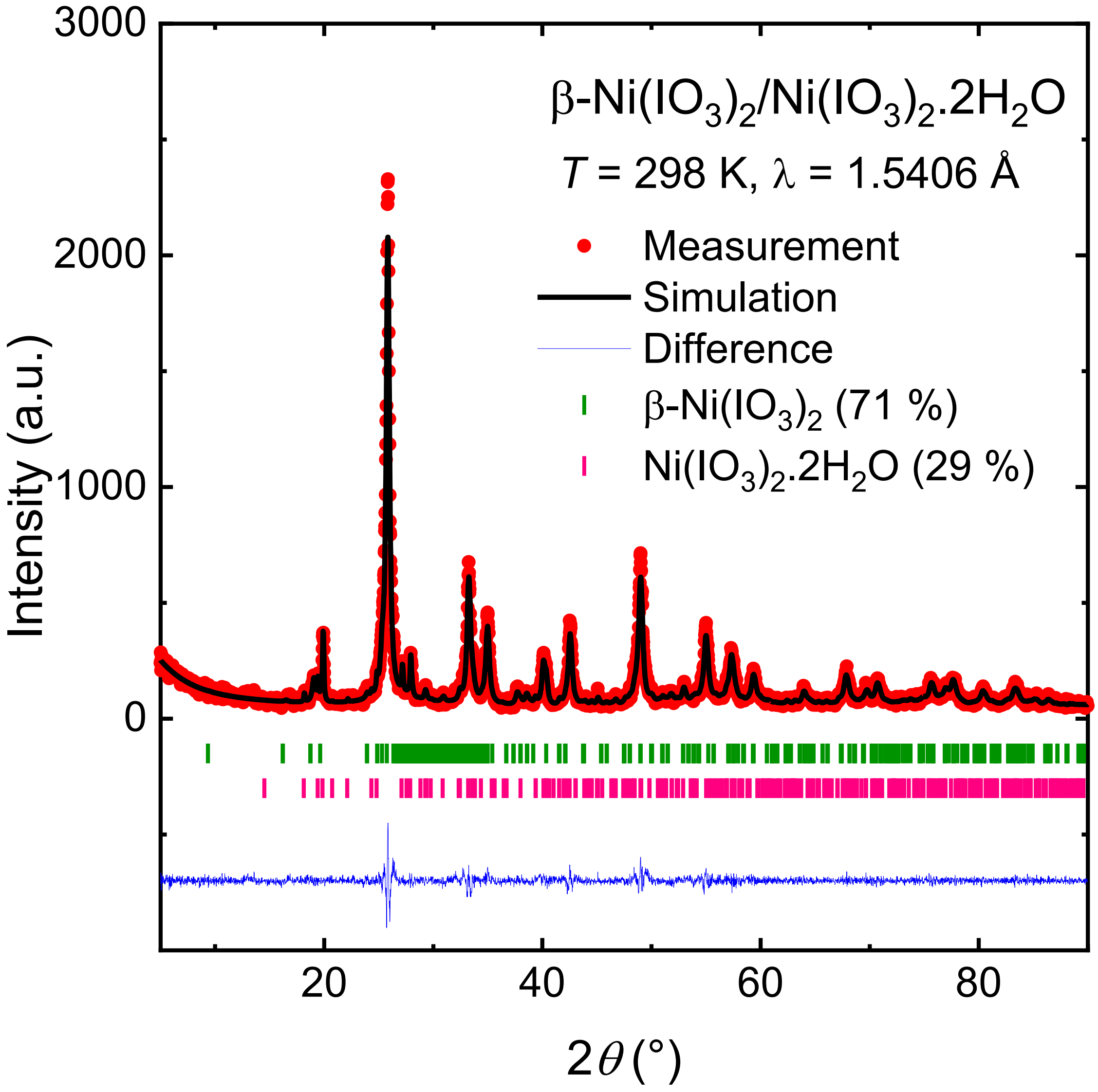


**Figure S1.** Rietveld fit (black line) of powder XRD pattern of the bulk polycrystalline β-$Ni(IO_3)_2$ precursor - β-$Ni(IO_3)_2$/$Ni(IO_3)_2.2H_2O$ mixture (red points) and the blue line is the difference between the measured data and the simulation.

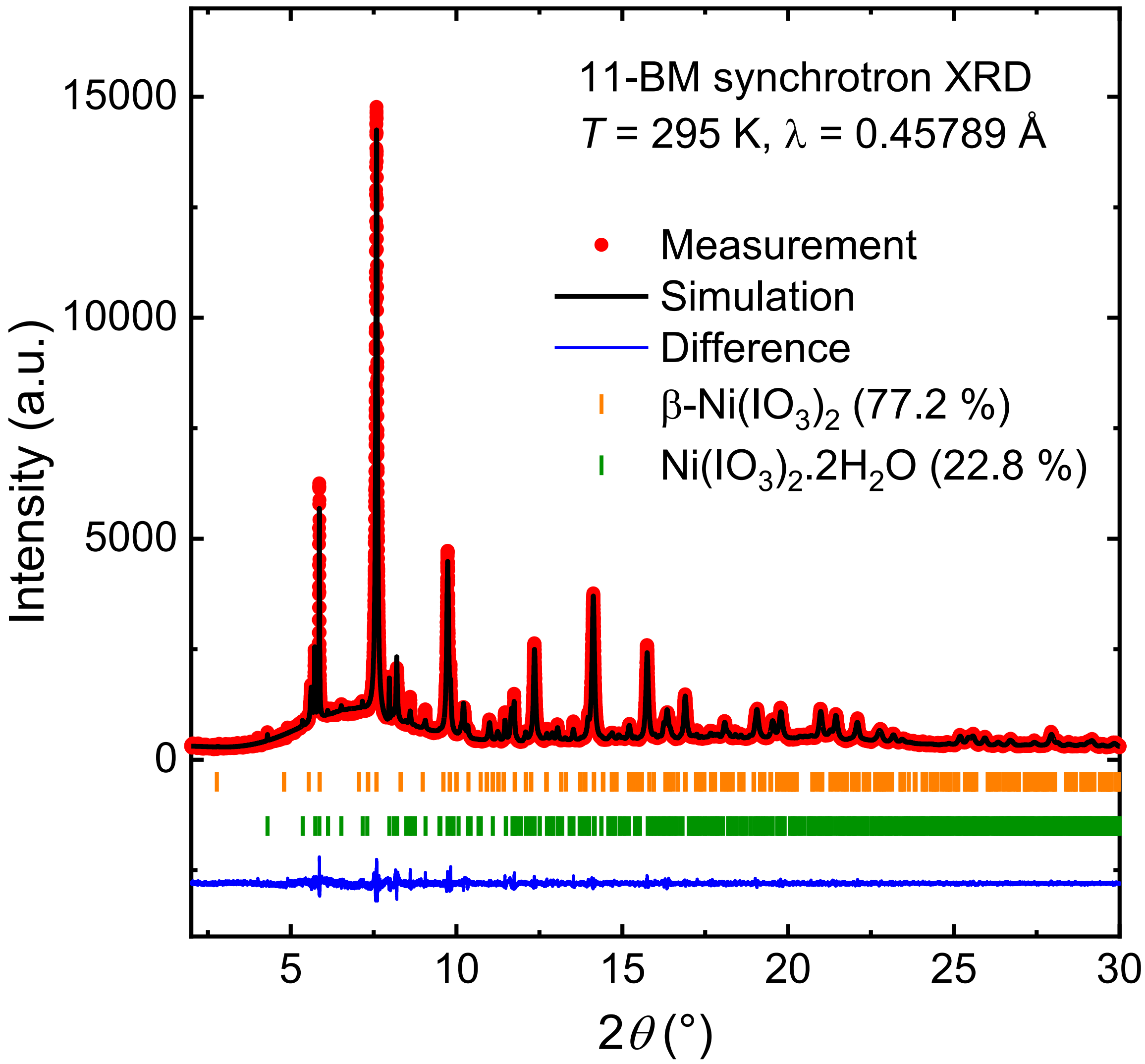


**Figure S2.** Rietveld fit (black line) of synchrotron XRD pattern of the bulk polycrystalline β-$Ni(IO_3)_2$ after 6 months in air (red points) and the blue line is the difference between the measured data and the simulation.

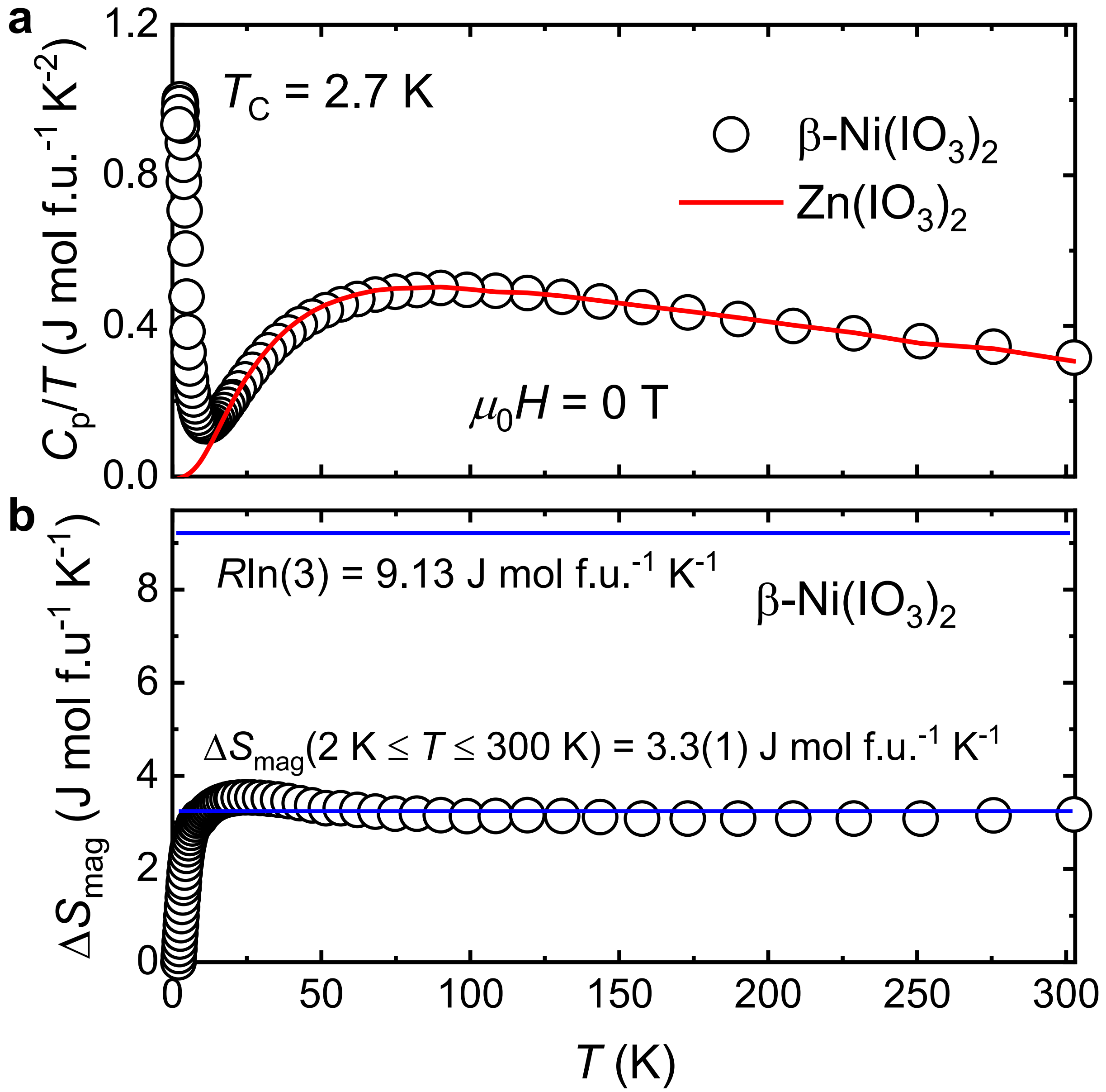


**Figure S3.** (a) Molar heat capacity over temperature ($C_p/T$) vs. temperature for β-$Ni(IO_3)_2$. (b) Magnetic entropy change from 2 K to 300 K, $\Delta S_{mag}$ (2 K ≤ $T$ ≤ 300 K) = 3.3(1) J mol$^{-1}$ K$^{-1}$ ~ 36 % of expected value for $S$ = 1 spins ($R$ln3).

**Table S1.** Crystal structure information and refinement parameters for β-$Ni(IO_3)_2$ obtained by Rietveld refinement of powder X-ray diffraction patterns.

| | Powder XRD |
|---|---|
| Chemical formula | $Ni(IO_3)_2$ |
| Formula weight (g/mol) | 408.51 |
| Temperature (K) | 298 |
| X-ray radiation | Cu $K_\alpha$ |
| Wavelength ($\lambda$, Å) | 1.5406 |
| Crystal system | Monoclinic |
| Space group | $P2_1$ |
| Z | 4 |
| $a$ (Å) | 10.8059(5) |
| $b$ (Å) | 5.1535(4) |
| $c$ (Å) | 10.7862(6) |
| $\alpha = \gamma$ (°) | 90 |
| $\beta$ (°) | 120.015(6) |
| $V$ (Å$^3$) | 520.11(4) |
| $\rho_{calc}$ (g/cm$^3$) | 5.217 |
| $2\theta$ (°) | 5 – 90 |
| GOF | 1.480 |
| $R$-factor (%) | $R_p{}^a = 6.61$, $R_{wp}{}^b = 9.84$, $R_{exp}{}^c = 10.78$ |

$$^aR_p = \sum_i^n \frac{|Y_i^{obs} - Y_i^{calc}|}{\sum_i^n Y_i^{obs}} \times 100\%$$

$$^bR_p = \sum_i^n \left(\frac{w_i\left(Y_i^{obs} - Y_i^{calc}\right)^2}{\sum_i^n Y_i^{obs}}\right)^{\frac{1}{2}} \times 100\%$$

$$^cR_{exp} = \left(\frac{n-p}{\sum_i^n w_i(Y_i^{obs})^2}\right)^{\frac{1}{2}} \times 100\%$$

**Table S2.** Atomic positions for β-$Ni(IO_3)_2$ obtained from the single crystal XRD.

| Atomic positions (Single crystal XRD) | | | | |
|---|---|---|---|---|
| | *x* | *y* | *z* | *U* ($Å^2$) |
| Ni1 | 0.4950(4) | 0.3880(9) | 0.2441(4) | 0.0089(10) |
| Ni2 | 0.0156(6) | 0.8942(13) | 0.2561(6) | 0.0224(12) |
| I1 | 0.33846(19) | 0.3010(4) | 0.43419(18) | 0.0081(7) |
| I2 | 0.6708(2) | 0.8059(4) | 0.09926(17) | 0.0112(8) |
| I3 | 0.8397(2) | 0.3150(4) | 0.4118(2) | 0.0126(8) |
| I4 | 0.1725(2) | 0.7959(5) | 0.0756(3) | 0.0168(8) |
| O1 | 0.636(3) | 0.602(6) | 0.212(3) | 0.017(5) |
| O2 | 0.471(3) | 0.104(8) | 0.578(4) | 0.023(6) |
| O3 | 0.321(3) | 0.608(5) | 0.094(3) | 0.015(5) |
| O4 | 0.875(4) | 0.111(11) | 0.292(4) | 0.032(9) |
| O5 | 0.187(4) | 0.108(9) | 0.404(6) | 0.038(11) |
| O6 | 0.839(4) | 0.656(7) | 0.130(5) | 0.032(9) |
| O7 | 0.144(4) | 0.652(8) | 0.214(3) | 0.022(6) |
| O8 | 0.368(4) | 0.169(5) | 0.293(4) | 0.016(5) |
| O9 | 0.550(3) | 0.649(5) | -0.070(3) | 0.015(5) |
| O10 | 0.671(3) | 0.158(5) | 0.364(3) | 0.016(6) |
| O11 | 0.029(4) | 0.640(7) | -0.076(4) | 0.021(6) |
| O12 | 0.933(4) | 0.150(8) | 0.575(3) | 0.022(6) |

**Table S3.** Bond lengths and bond angles of β-$Ni(IO_3)_2$.

| **β-$Ni(IO_3)_2$** | | | |
|---|---|---|---|
| **Bond lengths (Å)** | | | |
| Ni1-O8 | 2.03(3) | I1-O5 | 1.79(4) |
| Ni1-O1 | 2.04(3) | I1-O2 | 1.81(3) |
| Ni1-O10 | 2.05(3) | I1-O8 | 1.84(3) |
| Ni1-O3 | 2.10(3) | I2-O1 | 1.78(3) |
| Ni1-O9 | 2.09(3) | I2-O6 | 1.84(4) |
| Ni1-O2 | 2.09(3) | I2-O9 | 1.82(3) |
| Ni2-O5 | 2.06(4) | I3-O12 | 1.76(3) |
| Ni2-O4 | 2.06(4) | I3-O10 | 1.82(3) |
| Ni2-O12 | 2.07(3) | I3-O4 | 1.85(4) |
| Ni2-O7 | 2.09(4) | I4-O11 | 1.79(4) |
| Ni2-O6 | 2.10(5) | I4-O3 | 1.79(3) |
| Ni2-O11 | 2.16(4) | I4-O7 | 1.83(3) |

**Table S4:** Calculated bond valence sum ($V_i$) of β-$Ni(IO_3)_2$.[1]

| **Ni1** | | **O1** | **O2** | **O3** | **O8** | **O9** | **O10** | **$V_i = \sum S_{ij}$** |
|---|---|---|---|---|---|---|---|---|
| | **$R_{ij}$** | 2.04(3) | 2.09(3) | 2.10(3) | 2.03(3) | 2.09(3) | 2.05(3) | |
| | **$S_{ij}$** | 0.30641 | 0.26498 | 0.25750 | 0.31558 | 0.26498 | 0.29755 | **1.7070** |
| | | | | | | | | |
| **Ni2** | | **O4** | **O5** | **O6** | **O7** | **O11** | **O12** | |
| | **$R_{ij}$** | 2.06(4) | 2.06(4) | 2.10(5) | 2.07(4) | 2.16(4) | 2.07(3) | |
| | **$S_{ij}$** | 0.28899 | 0.28899 | 0.25750 | 0.28072 | 0.21745 | 0.28072 | **1.6144** |
| | | | | | | | | |
| **I1** | | **O2** | **O5** | **O8** | | | | |
| | **$R_{ij}$** | 1.81(3) | 1.79(4) | 1.84(3) | | | | |
| | **$S_{ij}$** | 1.8366 | 1.96321 | 1.6641 | | | | **5.4610** |
| **I2** | | **O1** | **O6** | **O9** | | | | |
| | **$R_{ij}$** | 1.78(3) | 1.84(4) | 1.82(3) | | | | |
| | **$S_{ij}$** | 2.03033 | 1.66410 | 1.77688 | | | | **5.4713** |
| **I3** | | **O4** | **O10** | **O12** | | | | |
| | **$R_{ij}$** | 1.85(4) | 1.82(3) | 1.76(3) | | | | |
| | **$S_{ij}$** | 1.61085 | 1.77688 | 2.17275 | | | | **5.5605** |
| **I4** | | **O3** | **O7** | **O11** | | | | |
| | **$R_{ij}$** | 1.79(4) | 1.83(3) | 1.79(4) | | | | |
| | **$S_{ij}$** | 1.96321 | 1.71941 | 1.96321 | | | | **5.6458** |

The ideal oxidation state of Ni and I are +2 and +5 respectively.

**References**

1. Altermatt, D.; Brown, I. D., The automatic searching for chemical bonds in inorganic crystal structures. *Acta Crystallogr. B* **1985,** *41* (4), 240-244.
